# Design of Ni(OH)_2_/M-MMT Nanocomposite With Higher Charge Transport as a High Capacity Supercapacitor

**DOI:** 10.3389/fchem.2022.916860

**Published:** 2022-05-26

**Authors:** G. M. Xu, M. Wang, H. L. Bao, P. F. Fang, Y. H. Zeng, L. Du, X. L. Wang

**Affiliations:** ^1^ School of Mechanical Engineering, Liaoning Technical University, Fuxin, China; ^2^ School of Materials Science and Engineering, Liaoning Technical University, Fuxin, China; ^3^ Key Laboratory of Mineral High Value Conversion and Energy Storage Materials of Liaoning Province, Fuxin, China

**Keywords:** montmorillonite, Ni(OH)_2_, charge transport, nano-petal multilayered nanostructure, electrochemical performance

## Abstract

Nano-petal nickel hydroxide was prepared on multilayered modified montmorillonite (M-MMT) using one-step hydrothermal method for the first time. This nano-petal multilayered nanostructure dominated the ion diffusion path to be shorted and the higher charge transport ability, which caused the higher specific capacitance. The results showed that in the three-electrode system, the specific capacitance of the nanocomposite with 4% M-MMT reached 1068 F/g at 1 A/g and the capacity retention rate was 70.2% after 1,000 cycles at 10 A/g, which was much higher than that of pure Ni(OH)_2_ (824 F/g at 1 A/g), indicating that the Ni(OH)_2_/M-MMT nanocomposite would be a new type of environmentally friendly energy storage supercapacitor.

## Introduction

With the vigorous development of science and technology, there appear many problems, such as: energy crisis, environmental pollution, greenhouse effect, etc., (Shi et al., 2020; Delbari et al., 2021). It is now crucial to find new, low-cost and environmentally friendly energy conversion and storage systems (Liu et al., 2020). Until now, a great deal of research had been done on energy storage materials and systems (Duraković and Mešetović, 2019; Fleischmann et al., 2020; Dou et al., 2021; Yang et al., 2021; Caturwati et al., 2022; Lv et al., 2022).

Supercapacitors had been widely used in modern energy storage devices due to their higher power density, fast charging and discharging speed, and low impact on the ecological environment, which have become a hot research issue for many researchers (Wiston and Ashok, 2019; Ovhal et al., 2020; Wang et al., 2021a; Zhang et al., 2021). Supercapacitors can be classified into pseudocapacitor (PCs), electric double layer supercapacitors (EDLCs), and hybrid supercapacitors (HCs) according to different storage mechanisms. Carbon-based materials such as activated carbon (Wang et al., 2021b), graphene (Wan et al., 2020), carbon nanotubes (Saikia et al., 2020), etc., had been used for EDLC. On the other hand, transition metal oxides/hydroxides and conducting polymers were widely used in pseudocapacitors, which had higher power densities than EDLCs. Several metal oxides are used as electrode materials, such as RuO_2_, NiO, CuO, MnO_2_, TiO_2_, etc., (Zheng et al., 2018). Metal hydroxides were an attractive alternative as electrode materials for higher-energy and higher-power supercapacitors because of the higher specific capacitance and the higher charge transport ability, which may make the construction of higher-energy, higher-power supercapacitors more feasible (Soserov et al., 2018a).

Nickel hydroxide had the characteristics of being the cheapest, high specific capacity and good stability among electrode materials, and had become an important supercapacitor electrode material. The preparation and performance have become one of the current research hotspots (Xing et al., 2012; Liu et al., 2017; Wu et al., 2020; Yang et al., 2020). Due to its low specific surface area and poor electrical conductivity, the diffusion distance of the electrolyte during charging is very short, and often only the surface part of the active material exchanges charges, and a large amount of internal space does not participate in the electrochemical energy storage process (Singh et al., 2017). There appears Ni(OH)_2_ aggregation formation during the preparation process, causing poor electrochemical performance (Soserov et al., 2018b). To solve the above problems, many scholars have loaded metal hydroxides on carbon materials with high specific surface area and high electrical conductivity or composited with metal oxides to improve the capacitance performance and cycle stability (Li et al., 2017; Liang et al., 2017; Chen et al., 2020; Li et al., 2020; Rawat et al., 2022). MMT was a layered substance composed of three parts, that is, two silicon-oxygen tetrahedra are located on the upper and lower sides of symmetry, and an aluminum-oxygen octahedron is sandwiched in the middle. These three parts constitute the 2:1 structural configuration of the entire montmorillonite crystal. MMT had a broad interlayer domain, which was very effective in storing and adsorbing water and organic matter, and also facilitated charge transport, so it was feasible to apply it in supercapacitors (Li et al., 2021a).

Many researchers on the utilization of montmorillonite were in the fields of environment, catalysis and biology, but very little in the field of supercapacitors (Numan et al., 2017). Therefore, in this paper, we designed of Ni(OH)_2_/M-MMT nanocomposite with higher charge transport as a high capacity supercapacitor for the first time, and it is also the first time that the experiment and computational simulation of montmorillonite were combined. The electrode material was prepared by a simple hydrothermal process without using alkalis, organic solvents and chemical binders. Compared with pure Ni(OH)_2_ nanomaterials, the prepared Ni(OH)_2_/M-MMT nanocomposite would have higher capacitance and specific surface area and have great potential in the field of supercapacitors.

## Experiment

### Preparation of Modified Montmorillonite

5 g montmorillonite was dissolved in 50 ml deionized water and stirred for 30 min Na_2_CO_3_ powder was added, and then stirred to full reaction, the PH value of the solution was reached a weak alkaline with NaOH and HCl, continue to stir for 2 h, after the stirring is over, use deionized water for suction filtration, wash three times, put it in a drying box for 6 h, and finally grind the dried sample through a 300-mesh fine sieve. So far, the modified montmorillonite has been prepared.

### Preparation of Ni(OH)_2_/M-MMT Nanocomposite

**FIGURE 1:**
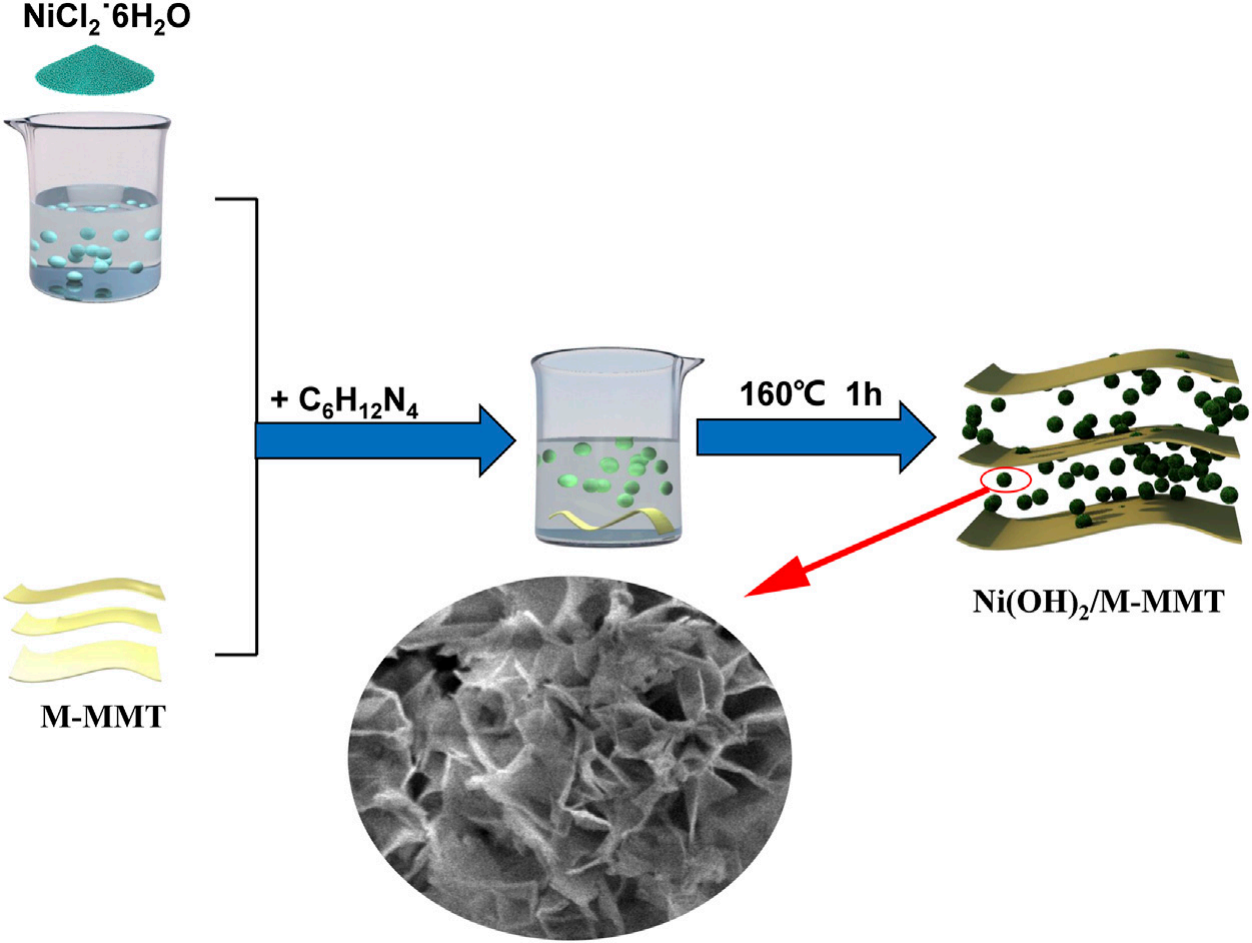
The flow chart of the preparation of Ni(OH)_2_/M-MMT nanocomposite.

8 mmoL NiCl_2_˙6H_2_O was dissolved in 30 ml deionized water. After NiCl_2_˙6H_2_O is completely dissolved in deionized water, modified montmorillonite with different contents (2, 4, 6, 8%) was added and stirred. After 1 h, 3 mmoL of hexamethylenetetramine was added, mixed and stirred for 30 min. The solution was added to the reaction kettle and reacted at 160°C for 1 h. After suction filtration, washing, drying, and finally grinding into powder. The whole experimental process was shown in Figure 1.

### Material Characterization

The micro-morphology of the composite material was observed by SEM(JSM-7500F). The phase analysis and structural characterization of the Ni(OH)_2_/M-MMT were carried out by XRD (SHIMADZU XRD-6100). The bonding condition of the nanocomposite was analyzed by XPS (SHIMADZU/KRATOS AXIS Ultra DLD). The specific surface area and pore distribution of the sample were measured by ASAP 2460 BET surface analyzer.

### Electrochemical Properties Measurement

The electrochemical performance of the three-electrode system was tested in 1 mol/L KOH electrolyte with sample electrode as working electrode, mercuric oxide electrode as reference electrode and platinum sheet as counter electrode, using Shanghai Chenhua CHI660E electrochemical workstation.

### Computational Method

First-principle calculation of Ni(OH)_2_/MMT nanocomposite microstructure model was carried out by CASTEP module of Materials Studio calculation software. The interaction between adsorbates and different surfaces was accurately described using Perdew-Burke-Ernzerhof (PBE) function under generalized gradient approximation (GGA). During the whole calculation process, the number of plane wave basis functions was determined by the kinetic energy cut-off point Ecut, which was selected as 400 eV. The effects of various nuclei and inner electrons on outer electrons were described by ultra-soft pseudopotentials. 3 × 3 × 1 k-point grid was used for Brillouin zone integration. A single cell material model with 8 H atoms, 32 O atoms, 4 Al atoms, 8 Si atoms and 4 Ni atoms were constructed.

## Results and Discussion

**FIGURE 2:**
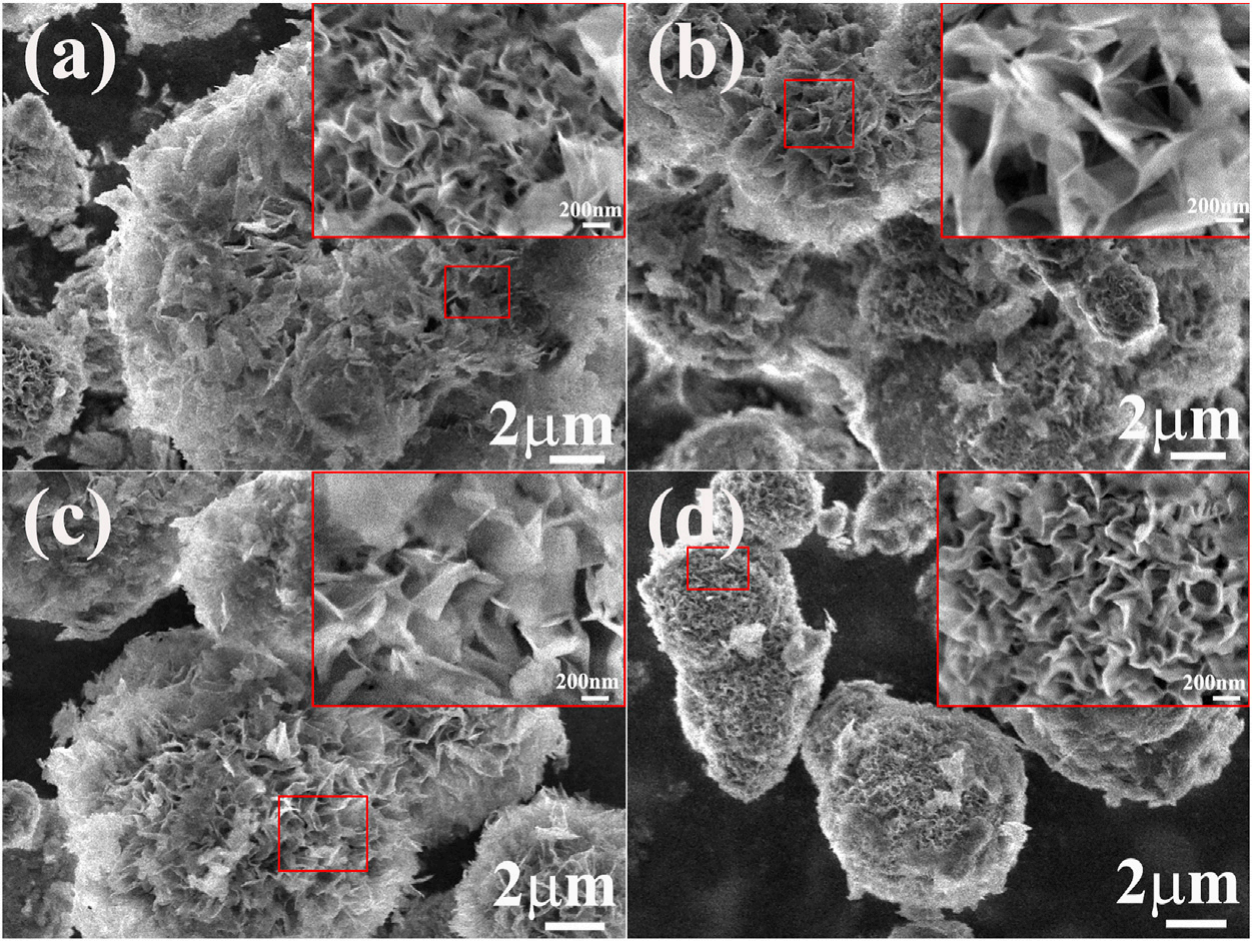
SEM morphology of Ni(OH)_2_/M-MMT nanocomposite with different M-MMT content. **(A)** M-MMT∼2%, **(B)** M-MMT∼4%, **(C)** M-MMT∼6%, **(D)** M-MMT∼8% (inset: locally enlarged image).

The SEM images of the Ni(OH)_2_/M-MMT nanocomposite with different content was shown in Figure 2, the inset image was locally enlarged. EDS pattern was showed in the (Supplementary Figure S1). From EDS analysis and the content of each element of the Ni(OH)_2_/M-MMT nanocomposite, it can be seen that the contents of O, Ni, Al and Si were 65.4%, 34.4%, 0.05% and 0.15%, respectively. With the increase of M-MMT content, the nano-petal morphology of the Ni(OH)_2_/M-MMT nanocomposite persisted and was not destroyed due to the influence of the reaction conditions. At high magnification, it was obvious that the ultrathin nanoplates grown vertically or attached obliquely on M-MMT and were interlaced with each other to form a highly open nano-petals structure, in which the layered-space of nano-petals became wider as M-MMT content increased from 2 to 4% (see inset of Figures 2A,B), and the layer thickness and layer spacing of the nanocomposite with 4% M-MMT was about 23.0 and 68.30 nm respectively. The nano-petals of Ni(OH)_2_/M-MMT nanocomposite appeared larger and became looser at the addition of 4%, which can increase specific surface area, provide more electroactive surface sites, and the more charge transport. The spacing of the flowers is gradually reduced, and the vertically oriented nano-petals tend to cluster together to form micro-flowers. With increasing, the spacing of nano-petals of 4–8% nanocomposite gradually decreased. This was because when the Ni(OH)_2_ entered the interlayer of the montmorillonite, with the increase of the M-MMT content, the nanoflowers “blooming” was inhibited, resulting in a decrease in the interlayer spacing of the Ni(OH)_2_ nano-petals.

**FIGURE 3:**
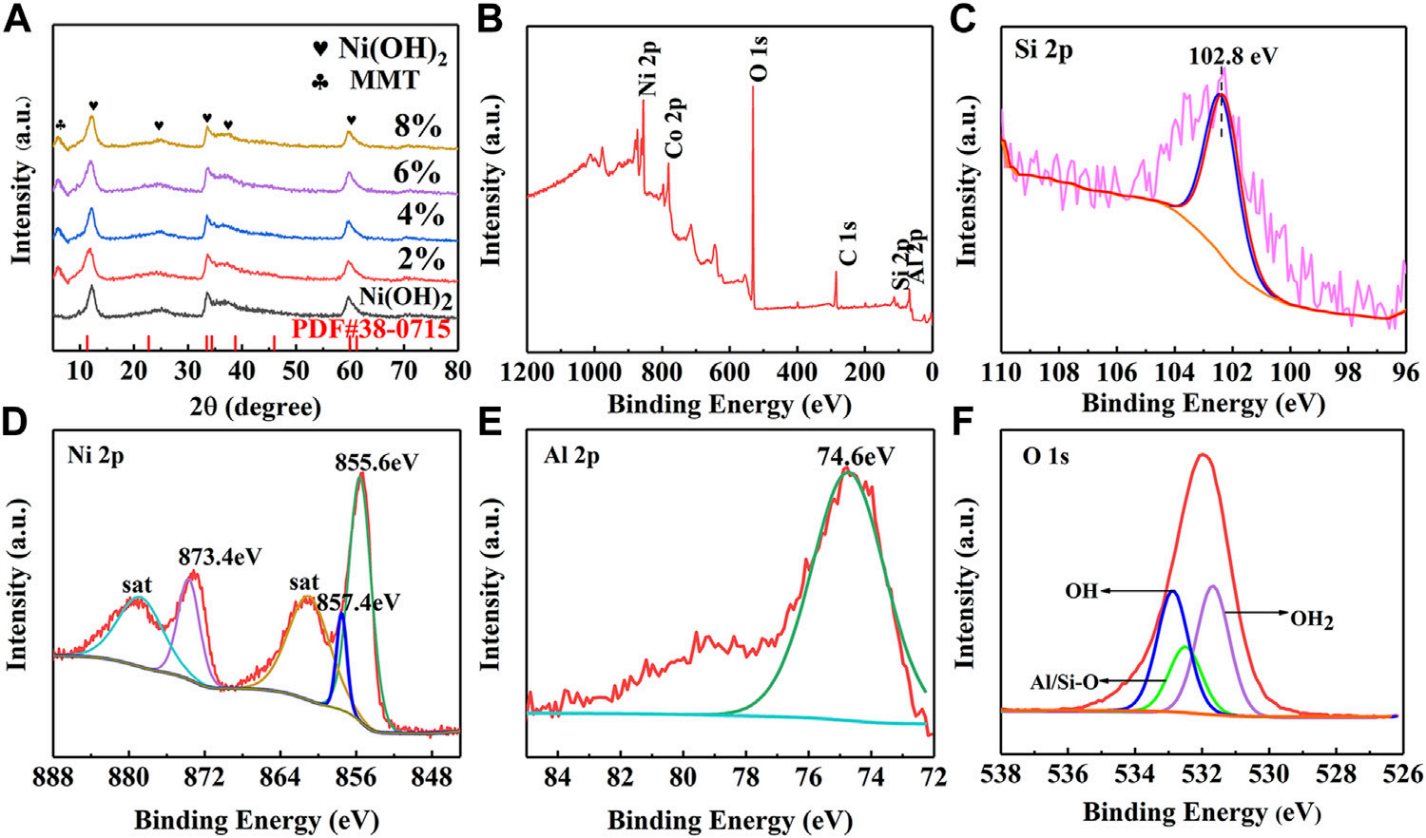
**(A)** XRD patterns of pure Ni(OH)_2_ and Ni(OH)_2_/M-MMT nanocomposite with different content, **(B)** XPS full survey spectra of the Ni(OH)_2_/M-MMT nanocomposite, XPS spectra of **(C)** Si 2p, **(D)** Ni 2p, **(E)** Al 2p and **(F)** O 1s for Ni(OH)_2_/M-MMT nanocomposite.

XRD patterns of Ni(OH)_2_/M-MMT nanocomposite with different ratios was shown in Figure 3A. It was found that the diffraction peaks of M-MMT were located at 7.15^°^, 19.83^°^, 28.43^°^, 35.16^°^, and 61.76^°^, corresponding to (001) (100) (005) (110) and (300), respectively, which was consistent with the standard card of montmorillonite (JCPDS#12-0,204) (Ge et al., 2021). The diffraction peaks of Ni(OH)_2_ located at 11.2^°^, 22.6^°^, 33.44^°^, 38^°^, 59.56^°^, and 61^°^. The peaks correspond to (003) (006) (101) (015) (110) and (113), respectively, which the XRD pattern matched the α-nickel hydroxide of JCPDS card number 38–0,715 (Zuo et al., 2020). The characteristic diffraction peaks of MMT and Ni(OH)_2_ appeared in Ni(OH)_2_/M-MMT, the diffraction peak of MMT was weaker than that of Ni(OH)_2_. The broad and weak diffraction peaks of Ni(OH)_2_/M-MMT nanocomposite indicate that the material was lower in crystallinity. However, the (003) diffraction peak of the Ni(OH)_2_/M-MMT nanocomposite was slightly shifted to the high-angle direction. This may be because the atomic radius of Si element in MMT was smaller than that of Ni. Therefore, the lattice of α-Ni(OH)_2_ shrank slightly after recombination. No peaks of other impurity phases could be found, indicating that the synthesized Ni(OH)_2_/M-MMT had high purity.

The X-ray photoelectron spectroscopy (XPS) full survey spectra of the Ni(OH)_2_/M-MMT nanocomposite was shown in Figure 3B and the corresponding deconvolution spectra of Si 2p, Ni 2p, Al 2p, O 1s was shown in Figures 3C–F, respectively. The peak at 102.8 eV in the energy spectrum of Si 2p in Figure 3C was characteristic of Si-O bonds in the montmorillonite structure (Payne et al., 2012; Biesinger et al., 2012; Cao et al., 2019). From Figure 3D Ni 2p scans depict spin-orbit peaks at 872.9 and 855.6 eV at chiral separation energy of 17.3 eV and two adjacent satellite vibrational peaks, assigned to Ni 2p_1/2_ and Ni 2p_3/2_ (He et al., 2022), indicating that Ni^2+^ existed in Ni(OH)_2_/M-MMT nanocomposite (Jiang et al., 2019). It can be seen that the energy spectrum of the Al 2p in Figure 3E element exhibits a characteristic peak with a binding energy of 74.6 eV. The exfoliated structure can be attributed to Al_2_O_3_ or AlO(OH) (Qin et al., 2015; Zhou et al., 2019). The peak located at 531.7 eV was usually associated with the hydroxide species (Bai et al., 2015). The lattice oxygen in silicon/aluminum oxides (Si/Al-O) was found with a binding energy of 532.5 eV (Yao et al., 2020). The contribution at 532.9 eV corresponded to physically adsorbed and chemically ad-sorbed water on or near the surface (Tong et al., 2016).

**FIGURE 4:**
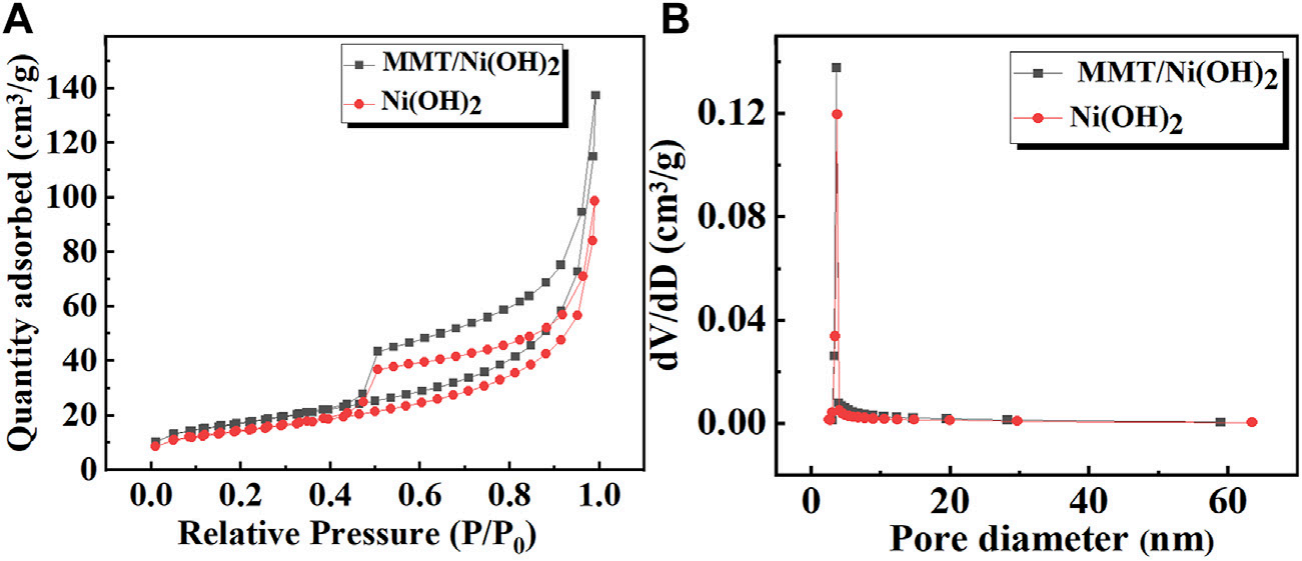
**(A)** N_2_ adsorption/desorption isotherms curves of pure Ni(OH)_2_ and Ni(OH)_2_/M-MMT nanocomposite, **(B)** Pore size distribution of pure Ni(OH)_2_ and Ni(OH)_2_/M-MMT nanocomposite.

In order to determine the specific surface area and pore size distribution of the prepared specimen, N_2_ adsorption/desorption isotherms BET analysis of Ni(OH)_2_ and Ni(OH)_2_/MMT nanocomposite was shown in Figure 4A. It was seen that the specific surface area of Ni(OH)_2_/M-MMT nanocomposite was 60 m^2^/g, which was higher compared with pure Ni(OH)_2_ (50 m^2^/g). All samples exhibited type IV isotherms, which were typical features of mesoporous materials (Yin et al., 2016). According to the pore size distribution (Figure 3B), the mean pore sizes of pure Ni(OH)_2_ and Ni(OH)_2_/M-MMT was 12.18 and 15.06 nm, respectively. The large specific surface area and moderate pore size of Ni(OH)_2_/M-MMT can expose more electroactive sites, provide more electroactive surface sites and the more charge transport.

**FIGURE 5:**
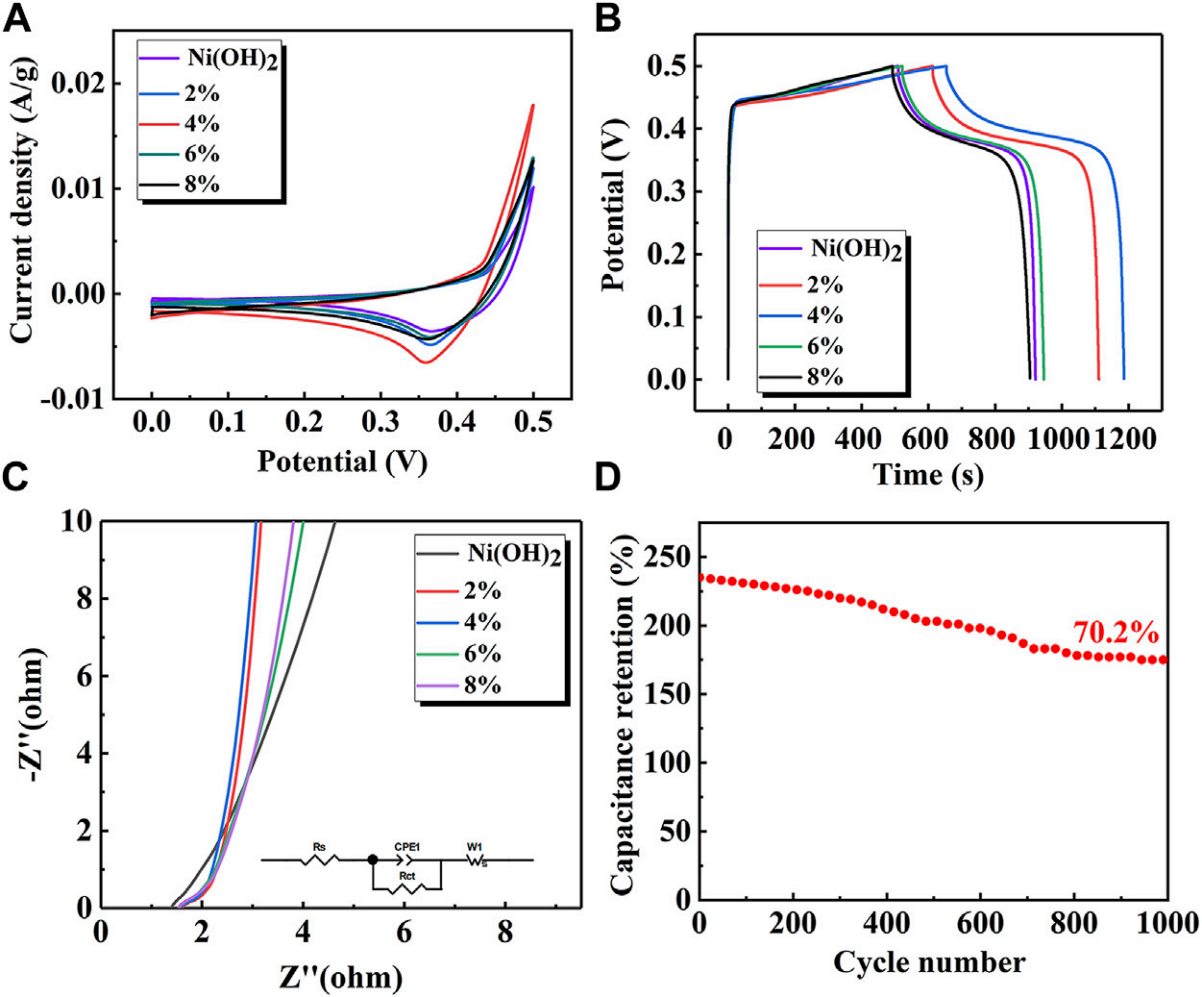
Electrochemical performance curves of pure Ni(OH)_2_ and Ni(OH)_2_/M-MMT, **(A)** Cyclic voltammetry curve, **(B)** Galvanostatic discharge curve, **(C)** The EIS (AC impedance) diagram of Ni(OH)_2_/M-MMT, **(D)** Long cycle curves of nanocomposite with M-MMT 4%.

The electrochemical performance of the Ni(OH)_2_ and Ni(OH)_2_/M-MMT nanocomposite with different M-MMT content was shown in Figure 5. Figure 5A showed the CV curves of Ni(OH)_2_ and Ni(OH)_2_/M-MMT nanocomposite with different M-MMT content. It can be seen that a pair of redox peaks caused by the redox reaction of Ni was generated and the pseudocapacitance characteristics was exhibited (Li et al., 2021b). The CV curve area of the Ni(OH)_2_/M-MMT nanocomposite with the M-MMT content of 4% was the largest, which indicated the capacitance of Ni(OH)_2_/M-MMT nanocomposite with the M-MMT content of 4% was larger than that of other M-MMT content, because the capacitance of the electrode was related to the integral CV curve area (Zheng et al., 2021).

The GCD curve of Ni(OH)_2_ and Ni(OH)_2_/M-MMT nanocomposite at 1 A/g was shown in Figure 5B. The platform indicated that this was a Faradaic reaction, in which the specific capacitance of pure Ni(OH)_2_ was 824 F/g, the specific capacitance of pure M-MMT was only 1.4 F/g (Supplementary Figure S2), but the specific capacitance of Ni(OH)_2_/M-MMT nanocomposite with different M-MMT content was 1000 F/g (2%), 1068 F/g (4%), 848 F/g (6%), 827.2 F/g (8%), respectively. It can be found that the specific capacitance of Ni(OH)_2_/M-MMT with 4% Ni(OH)_2_ had been improved with the addition of M-MMT. The specific capacitance of Ni(OH)_2_/M-MMT nanocomposite with 4% was increased to 130% compared with that of pure Ni(OH)_2_. This was because M-MMT had a multilayered “hamburger” structure, which provided greater ion exchange space, promoted ion diffusion path and increased the rate of charge transport. The nano-petals Ni(OH)_2_ entered the M-MMT interlayer during hydrothermal synthesis. The nano-petals surface had grooves and gap, which improved the movement of electrolyte ions. The gap between the petals facilitated the insertion of electrolyte ions into the electrode surface (Jiang et al., 2021). This enhanced the electrochemical activity of Ni(OH)_2_/M-MMT nanocomposite, resulting in a significant increase in the specific capacitance of the nanocomposite.

Figure 5C showed AC impedance curves of pure Ni(OH)_2_ and Ni(OH)_2_/M-MMT nanocomposite with different M-MMT content and the inset image was equivalent circuit diagram. The EIS data further validated the ion diffusion and conductance kinetics. The charge transfer resistance of the electrode material corresponds to the diameter of the semicircle in the high frequency range and lower resistance availed charge faster transport. The linear characteristic in the low frequency range represents the diffusion resistance of electrolyte ions on the surface. The smaller particle size and many interfaces of electrode material were beneficial to the diffusion of electrolytes (Ramesh et al., 2021). When the slope was closer to 90, the ion diffusion effect was stronger (Zou et al., 2020). From Figure 5C, the slope of Ni(OH)_2_/M-MMT nanocomposite in the low frequency range was larger than that of pure Ni(OH)_2_, and the slope of Ni(OH)_2_/M-MMT nanocomposite with 4% M-MMT was the highest in the low frequency range. This was because the layer spacing of M-MMT was larger, which increased the space of ion transmission and enhanced the conductivity.

The long cycle performance of the Ni(OH)_2_/M-MMT nanocomposite with 4% M-MMT at 10 A/g for 1,000 cycles was shown in Figure 5D. The specific capacitance was 175 F/g at 10 A/g after 1,000 cycles, and the capacitance retention rate reached 70.2%.

**FIGURE 6:**
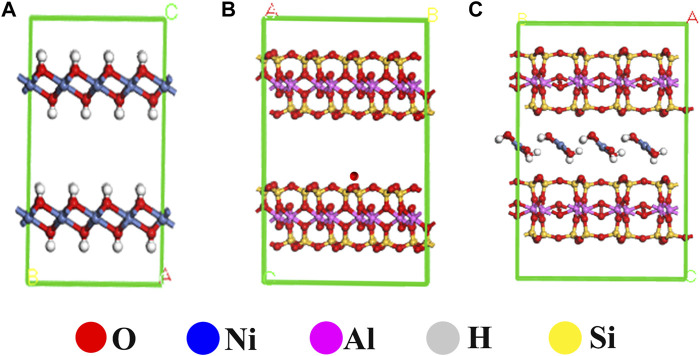
The structural optimization diagrams of **(A)** MMT, **(B)** Ni(OH)_2_ and **(C)** Ni(OH)_2_/MMT.

**FIGURE 7:**
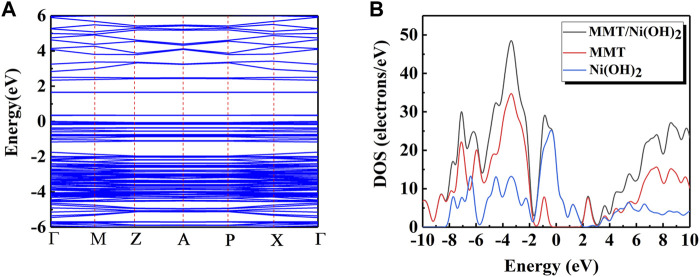
**(A)** The energy band diagram of Ni(OH)_2_/MMT; **(B)** The density of states of MMT, Ni(OH)_2_ and Ni(OH)_2_/MMT.

The structural model of MMT, Ni(OH)_2_, and Ni(OH)_2_/MMT was optimized based on the density functional theory (DFT) by Materials Studio (Boek et al., 1995; Tao et al., 2020; Han et al., 2021), as shown in Figure 6. Figure 7 showed the energy bands and density of states of Ni(OH)_2_/MMT nanocomposite. From Figure 7A, E_f_ = 0 was considered as the Fermi level, and the integration path was Г-M-Z-A-P-X-Г. The top of the valence band was close to the Fermi level, and its band gap value was 0.335 eV, which was a typical semiconductor feature (Kong et al., 2021).

To obtain a further understanding of the interaction between Ni(OH)_2_/MMT nanocomposite, the partial density of states (PDOS) and total density of states (TDOS) of Ni(OH)_2_/MMT nanocomposite were calculated and compared with that of Ni(OH)_2_ and MMT as shown in Figure 7B. In the Ni(OH)_2_/MMT system, the atoms of the Ni(OH)_2_/MMT nanocomposite material can gain more charges from Ni(OH)_2_. Based on the above calculation results, it was shown that the addition of MMT leaded to the obvious charge transfer of Ni^2+^, which increased the charge transport rate. At the Fermi level, the energy of the Ni(OH)_2_/MMT nanocomposite was higher than that of pure Ni(OH)_2_ and MMT, and the energy of Ni(OH)_2_ was higher than that of MMT, indicating that the energy of the nanocomposite mainly from Ni(OH)_2_ (Zhao et al., 2019). The state at the Fermi level was almost flat, indicating that the Ni(OH)_2_/MMT nanocomposite was in a stable state, which was consistent with the previous experiments and achieved the expected effect.

## Conclusion

1) The Ni(OH)_2_ nano-petal was grown on the modified montmorillonite, and the nano-petal was uniformly distributed and had a large specific surface area.
2) The specific capacitance of Ni(OH)_2_/M-MMT nanocomposite was 1086 F/g at 1 A/g under three electrodes, the capacitance retention rate of Ni(OH)_2_/M-MMT nanocomposite can reach 70.2% at 10 A/g after 1,000 cycles.
3) According to the first-principles calculation, the band gap value of the Ni(OH)_2_/MMT nanocomposite was 0.335 eV, and the state at the Fermi level was almost flat, indicating that the Ni(OH)_2_/MMT nanocomposite was in a stable state and presented a semiconductor structure. The Ni(OH)_2_/MMT nanocomposite could short the path of ion diffusion and improve the speed of charge transport.

## Data Availability

The original contributions presented in the study are included in the article/Supplementary Material, further inquiries can be directed to the corresponding author.

## References

[B1] Bai Y. Wang W. Wang R. Sun J. Gao L. (2015). Controllable Synthesis of 3D Binary Nickel-Cobalt Hydroxide/graphene/nickel Foam as a Binder-free Electrode for High-Performance Supercapacitors. J. Mat. Chem. A 3 (23), 12530–12538. 10.1039/c5ta01804h

[B2] Biesinger M. C. Lau L. W. M. Gerson A. R. Smart R. S. C. (2012). The Role of the Auger Parameter in XPS Studies of Nickel Metal, Halides and Oxides. Phys. Chem. Chem. Phys. 14 (7), 2434–2442. 10.1039/c2cp22419d 22249653

[B3] Boek E. S. Coveney P. V. Skipper N. T. (1995). Molecular Modeling of Clay Hydration: A Study of Hysteresis Loops in the Swelling Curves of Sodium Montmorillonites. Langmuir 11 (12), 4629–4631. 10.1021/la00012a008

[B4] Cao J. Mei Q. Wu R. Wang W. (2019). Flower-like Nickel-Cobalt Layered Hydroxide Nanostructures for Super Long-Life Asymmetrical Supercapacitors. Electrochimica Acta 321, 134711. 10.1016/j.electacta.2019.134711

[B5] Caturwati N. K. Rosyadi I. Yusuf Y. Saputra E. T. (2022). Lauric Acid as an Energy Storage Material to Increase Distillation Solar Productivity in Indonesia. Mater. Sci. Forum 1057, 144–151. 10.4028/p-11m66k

[B6] Chen G. Wan H. Ma W. Zhang N. Cao Y. Liu X. (2020). Layered Metal Hydroxides and Their Derivatives: Controllable Synthesis, Chemical Exfoliation, and Electrocatalytic Applications. Adv. Energy Mat. 10 (11), 1902535. 10.1002/aenm.201902535

[B7] Delbari S. A. Ghadimi L. S. Hadi R. Farhoudian S. Nedaei M. Babapoor A. (2021). Transition Metal Oxide-Based Electrode Materials for Flexible Supercapacitors: A Review. J. Alloys Compd. 857, 158281. 10.1016/j.jallcom.2020.158281

[B8] Dou Q. Wu N. Yuan H. Shin K. H. Tang Y. Mitlin D. (2021). Emerging Trends in Anion Storage Materials for the Capacitive and Hybrid Energy Storage and beyond. Chem. Soc. Rev. 50 (12), 6734–6789. 10.1039/d0cs00721h 33955977

[B9] Duraković B. Mešetović S. (2019). Thermal Performances of Glazed Energy Storage Systems with Various Storage Materials: An Experimental Study. Sustain. Cities Soc. 45, 422–430.

[B10] Fleischmann S. Mitchell J. B. Wang R. Zhan C. Jiang D.-e. Presser V. (2020). Pseudocapacitance: from Fundamental Understanding to High Power Energy Storage Materials. Chem. Rev. 120 (14), 6738–6782. 10.1021/acs.chemrev.0c00170 32597172

[B11] Ge W. Ma Q. Ai Z. Wang W. Jia F. Song S. (2021). Three-dimensional Reduced Graphene Oxide/montmorillonite Nanosheet Aerogels as Electrode Material for Supercapacitor Application. Appl. Clay Sci. 206, 106022. 10.1016/j.clay.2021.106022

[B12] Han Z. Cui Y. Meng Q. He M. Yan X. (2021). The Effect of Inorganic Salt on the Mechanical Properties of Montmorillonite and its Mechanism: A Molecular Dynamics Study. Chem. Phys. Lett. 781, 138982. 10.1016/j.cplett.2021.138982

[B13] He P. Xiong Y. Chen Y. Liu M. Zhu J. Gan M. (2022). One-step Synthesis of Natural Montmorillonite/hematite Composites with Enhanced Persulfate Catalytic Activity for Sulfamethoxazole Degradation: Efficiency, Kinetics, and Mechanism. Environ. Res. 204, 112326. 10.1016/j.envres.2021.112326 34748776

[B14] Jiang D. B. Jing C. Yuan Y. Feng L. Liu X. Dong F. (2019). 2D-2D Growth of NiFe LDH Nanoflakes on Montmorillonite for Cationic and Anionic Dye Adsorption Performance. J. colloid interface Sci. 540, 398–409. 10.1016/j.jcis.2019.01.022 30665166

[B15] Jiang D. Zheng M. You Y. Li F. Yuan H. Zhang W. (2021). β-Ni(OH)2/nickel-cobalt Layered Double Hydroxides Coupled with Fluorine-Modified Graphene as High-Capacitance Supercapacitor Electrodes with Improved Cycle Life. J. Alloys Compd. 875, 159929. 10.1016/j.jallcom.2021.159929

[B16] Kong L. Tang H. Wang X. Lei Y. Li B. Chang K. (2021). Study on the *In Situ* Sulfidation and Electrochemical Performance of Spherical Nickel Hydroxide. Int. J. Hydrogen Energy 46 (58), 30079–30089. 10.1016/j.ijhydene.2021.06.124

[B17] Li J. Qiao J. Lian K. (2020). Hydroxide Ion Conducting Polymer Electrolytes and Their Applications in Solid Supercapacitors: A Review. Energy Storage Mater. 24, 6–21. 10.1016/j.ensm.2019.08.012

[B18] Li S. Yu C. Yang J. Zhao C. Zhang M. Huang H. (2017). A Superhydrophilic "nanoglue" for Stabilizing Metal Hydroxides onto Carbon Materials for High-Energy and Ultralong-Life Asymmetric Supercapacitors. Energy Environ. Sci. 10 (9), 1958–1965. 10.1039/c7ee01040k

[B19] Li X. Li M. Huang Z. Liang G. Chen Z. Yang Q. (2021). Activating the I0/I+ Redox Couple in an Aqueous I2-Zn Battery to Achieve a High Voltage Plateau. Energy Environ. Sci. 14 (1), 407–413. 10.1039/d0ee03086d

[B20] Li Z. He J. Ma H. Zang L. Li D. Guo S. (2021). Preparation of Heterogeneous TiO2/g-C3n4 with a Layered Mosaic Stack Structure by Use of Montmorillonite as a Hard Template Approach: TC Degradation, Kinetic, Mechanism, Pathway and DFT Investigation. Appl. Clay Sci. 207, 106107. 10.1016/j.clay.2021.106107

[B21] Liang C. Bao J. Li C. Huang H. Chen C. Lou Y. (2017). One-dimensional Hierarchically Porous Carbon from Biomass with High Capacitance as Supercapacitor Materials. Microporous Mesoporous Mater. 251, 77–82. 10.1016/j.micromeso.2017.05.044

[B22] Liu J. Hu R. Liu H. Ma J. (2017). Chips Assembled Cuboid-like Nickel hydroxide/rGO Composite Material for High Performance Supercapacitors. J. Alloys Compd. 718, 349–355. 10.1016/j.jallcom.2017.05.198

[B23] Liu S. Wei L. Wang H. (2020). Review on Reliability of Supercapacitors in Energy Storage Applications. Appl. Energy 278, 115436. 10.1016/j.apenergy.2020.115436

[B24] Lv J. Xie J. Mohamed A. G. A. Zhang X. Wang Y. (2022). Photoelectrochemical Energy Storage Materials: Design Principles and Functional Devices towards Direct Solar to Electrochemical Energy Storage. Chem. Soc. Rev. 51, 1511. 10.1039/D1CS00859E 35137737

[B25] Numan A. Duraisamy N. Saiha Omar F. Gopi D. Ramesh K. Ramesh S. (2017). Sonochemical Synthesis of Nanostructured Nickel Hydroxide as an Electrode Material for Improved Electrochemical Energy Storage Application. Prog. Nat. Sci. Mater. Int. 27 (4), 416–423. 10.1016/j.pnsc.2017.06.003

[B26] Ovhal M. M. Kumar N. Hong S.-K. Lee H.-W. Kang J.-W. (2020). Asymmetric Supercapacitor Featuring Carbon Nanotubes and Nickel Hydroxide Grown on Carbon Fabric: A Study of Self-Discharging Characteristics. J. Alloys Compd. 828, 154447. 10.1016/j.jallcom.2020.154447

[B27] Payne B. P. Biesinger M. C. McIntyre N. S. (2012). Use of Oxygen/nickel Ratios in the XPS Characterisation of Oxide Phases on Nickel Metal and Nickel Alloy Surfaces. J. Electron Spectrosc. Relat. Phenom. 185 (5-7), 159–166. 10.1016/j.elspec.2012.06.008

[B28] Qin D. Niu X. Qiao M. Liu G. Li H. Meng Z. (2015). Adsorption of Ferrous Ions onto Montmorillonites. Appl. Surf. Sci. 333, 170–177. 10.1016/j.apsusc.2015.02.019

[B29] Ramesh S. Karuppasamy K. Haldorai Y. Sivasamy A. Kim H.-S. Kim H. S. (2021). Hexagonal Nanostructured Cobalt Oxide @ Nitrogen Doped Multiwalled Carbon Nanotubes/polypyyrole Composite for Supercapacitor and Electrochemical Glucose Sensor. Colloids Surfaces B Biointerfaces 205, 111840. 10.1016/j.colsurfb.2021.111840 33992823

[B30] Rawat S. Mishra R. K. Bhaskar T. (2022). Biomass Derived Functional Carbon Materials for Supercapacitor Applications. Chemosphere 286, 131961. 10.1016/j.chemosphere.2021.131961 34426294

[B31] Saikia B. K. Benoy S. M. Bora M. Tamuly J. Pandey M. Bhattacharya D. (2020). A Brief Review on Supercapacitor Energy Storage Devices and Utilization of Natural Carbon Resources as Their Electrode Materials. Fuel 282, 118796. 10.1016/j.fuel.2020.118796

[B32] Shi J. Jiang B. Li C. Yan F. Wang D. Yang C. (2020). Review of Transition Metal Nitrides and Transition Metal Nitrides/carbon Nanocomposites for Supercapacitor Electrodes. Mater. Chem. Phys. 245, 122533. 10.1016/j.matchemphys.2019.122533

[B33] Singh S. Shinde N. M. Xia Q. X. Gopi C. V. V. M. Yun J. M. Mane R. S. (2017). Tailoring the Morphology Followed by the Electrochemical Performance of NiMn-LDH Nanosheet Arrays through Controlled Co-doping for High-Energy and Power Asymmetric Supercapacitors. Dalton Trans. 46 (38), 12876–12883. 10.1039/c7dt01863k 28920984

[B34] Soserov L. Stoyanova A. Boyadzhieva T. Koleva V. Kalapsazova M. Stoyanova R. (2018). Nickel-manganese Structured and Multiphase Composites as Electrodes for Hybrid Supercapacitors. Electrochimica Acta 283, 1063–1071. 10.1016/j.electacta.2018.06.191

[B35] Soserov L. Stoyanova A. Boyadzhieva T. Koleva V. Kalapsazova M. Stoyanova R. (2018). Nickel-manganese Structured and Multiphase Composites as Electrodes for Hybrid Supercapacitors. Electrochimica Acta 283, 1063–1071. 10.1016/j.electacta.2018.06.191

[B36] Tao E. Ma D. Yang S. Hao X. (2020). Graphene Oxide-Montmorillonite/sodium Alginate Aerogel Beads for Selective Adsorption of Methylene Blue in Wastewater. J. Alloys Compd. 832, 154833. 10.1016/j.jallcom.2020.154833

[B37] Tong X. Chen S. Guo C. Xia X. Guo X.-Y. (2016). Mesoporous NiCo2O4 Nanoplates on Three-Dimensional Graphene Foam as an Efficient Electrocatalyst for the Oxygen Reduction Reaction. ACS Appl. Mat. Interfaces 8 (42), 28274–28282. 10.1021/acsami.5b10044 26796978

[B38] Wan L. Chen D. Liu J. Zhang Y. Chen J. Du C. (2020). Facile Preparation of Porous Carbons Derived from Orange Peel via Basic Copper Carbonate Activation for Supercapacitors. J. Alloys Compd. 823, 153747. 10.1016/j.jallcom.2020.153747

[B39] Wang H. Wang M. Wang J. (2021). Nickel Silicate Hydroxide on Hierarchically Porous Carbon Derived from Rice Husks as High-Performance Electrode Material for Supercapacitors. Int. J. Hydrogen Energy 46 (71), 35351–35364. 10.1016/j.ijhydene.2021.08.062

[B40] Wang Y. Huang H. Choi W. M. (2021). Polypyrrole Decorated Cobalt Carbonate Hydroxide on Carbon Cloth for High Performance Flexible Supercapacitor Electrodes. J. Alloys Compd. 886, 161171. 10.1016/j.jallcom.2021.161171

[B41] Wiston B. R. Ashok M. (2019). Electrochemical Performance of Hydrothermally Synthesized Flower-like α-nickel Hydroxide. Vacuum 160, 12–17. 10.1016/j.vacuum.2018.11.014

[B42] Wu Y. Yang Y. Liu B. Hu M. Min X. Wu Y. (2020). Self-assembled Three-Dimension Flower-like Nickel Hydroxide Synthesis with One-Pot Hydrothermal Method for Electrochemical Applications. Mater. Lett. 264, 127358. 10.1016/j.matlet.2020.127358

[B43] Xing S. Wang Q. Ma Z. Wu Y. Gao Y. (2012). Synthesis of Mesoporous α-Ni(OH)2 for High-Performance Supercapacitors. Mater. Lett. 78, 99–101. 10.1016/j.matlet.2012.03.023

[B44] Yang L. Villalobos U. Akhmetov B. Gil A. Khor J. O. Palacios A. (2021). A Comprehensive Review on Sub-zero Temperature Cold Thermal Energy Storage Materials, Technologies, and Applications: State of the Art and Recent Developments. Appl. Energy 288, 116555. 10.1016/j.apenergy.2021.116555

[B45] Yang S.-B. Tsai Y.-C. Wu M.-S. (2020). Honeycomb-like Copper/cuprous Oxide with Supported Nickel Hydroxide Layer as an Electrode Material for Electrochemical Oxidation of Urea. J. Alloys Compd. 836, 155533. 10.1016/j.jallcom.2020.155533

[B46] Yao D. Shi Y. Pan H. Zhong D. Hou H. Wu X. (2020). Promotion Mechanism of Natural Clay Colloids in the Adsorption of Arsenite on Iron Oxide Particles in Water. Chem. Eng. J. 392, 123637. 10.1016/j.cej.2019.123637

[B47] Yin X.-m. Xie X.-m. Wu X. An X. (2016). Catalytic Performance of Nickel Immobilized on Organically Modified Montmorillonite in the Steam Reforming of Ethanol for Hydrogen Production. J. Fuel Chem. Technol. 44 (6), 689–697. 10.1016/s1872-5813(16)30033-0

[B48] Zhang M. Wang Y. Guo X. Li R. Peng Z. Zhang W. (2021). High-Performance Nickel Cobalt Hydroxide Nanosheets/Graphene/Ni Foam Composite Electrode for Supercapacitor Applications. J. Electroanal. Chem. 897, 115543. 10.1016/j.jelechem.2021.115543

[B49] Zhao Q. Fu L. Jiang D. Ouyang J. Hu Y. Yang H. (2019). Nanoclay-modulated Oxygen Vacancies of Metal Oxide. Commun. Chem. 2 (1), 11. 10.1038/s42004-019-0112-9

[B50] Zheng J. Lian X. Wu M. Zheng F. Gao Y. Niu H. (2021). One-step Preparation of Ni3S4 Quantum Dots Composite Graphene/carbon Nanotube Conductive Network for Asymmetric Supercapacitor. J. Alloys Compd. 859, 158247. 10.1016/j.jallcom.2020.158247

[B51] Zheng M. Tang H. Li L. Hu Q. Zhang L. Xue H. (2018). Hierarchically Nanostructured Transition Metal Oxides for Lithium‐Ion Batteries. Adv. Sci. 5 (3), 1700592. 10.1002/advs.201700592 PMC5867132 29593962

[B52] Zhou G. Wang Y. Zhou R. Wang C. Jin Y. Qiu J. (2019). Synthesis of Amino-Functionalized bentonite/CoFe2O4@MnO2 Magnetic Recoverable Nanoparticles for Aqueous Cd2+ Removal. Sci. Total Environ. 682, 505–513. 10.1016/j.scitotenv.2019.05.218 31129538

[B53] Zou S. Liu X. Xiao Z. Xie P. Liu K. Lv C. (2020). Engineering the Interface for Promoting Ionic/electronic Transmission of Organic Flexible Supercapacitors with High Volumetric Energy Density. J. Power Sources 460, 228097. 10.1016/j.jpowsour.2020.228097

[B54] Zuo H. Fu W. Fan R. Dastan D. Wang H. Shi Z. (2020). Bilayer Carbon Nanowires/nickel Cobalt Hydroxides Nanostructures for High-Performance Supercapacitors. Mater. Lett. 263, 127217. 10.1016/j.matlet.2019.127217

