# Supplementary figures and images for: Design of Ni(OH)2/M-MMT Nanocomposite With Higher Charge Transport as a High Capacity Supercapacitor
Source: Front Chem. 2022 May 26;10:916860. doi: 10.3389/fchem.2022.916860 (PMC9197183; doi:10.3389/fchem.2022.916860)

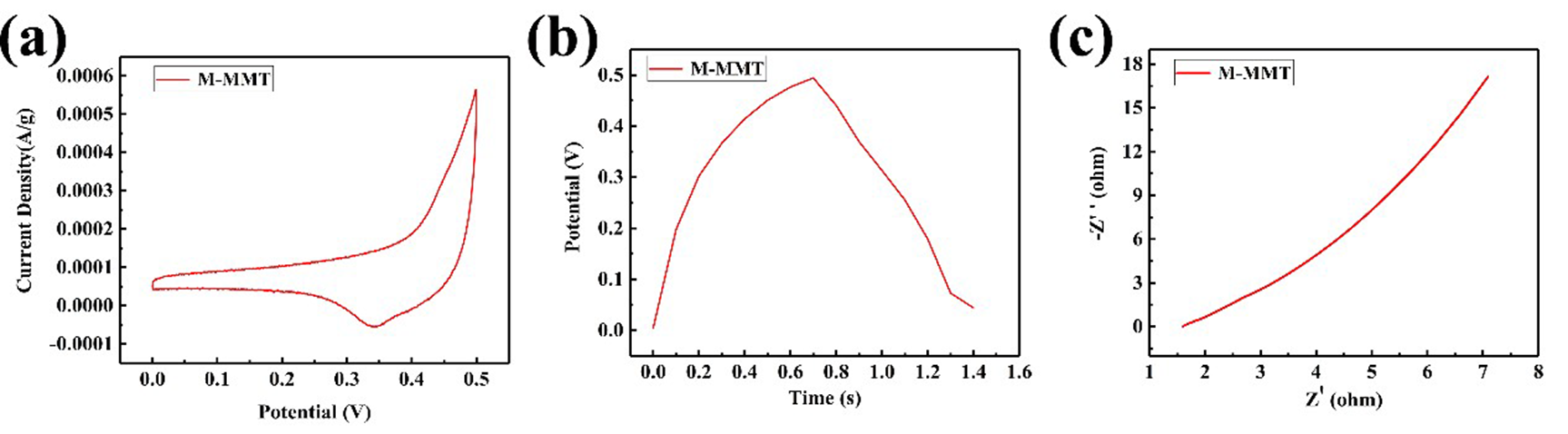

Supplement: Supplementary file 1 [file Image2.TIF]

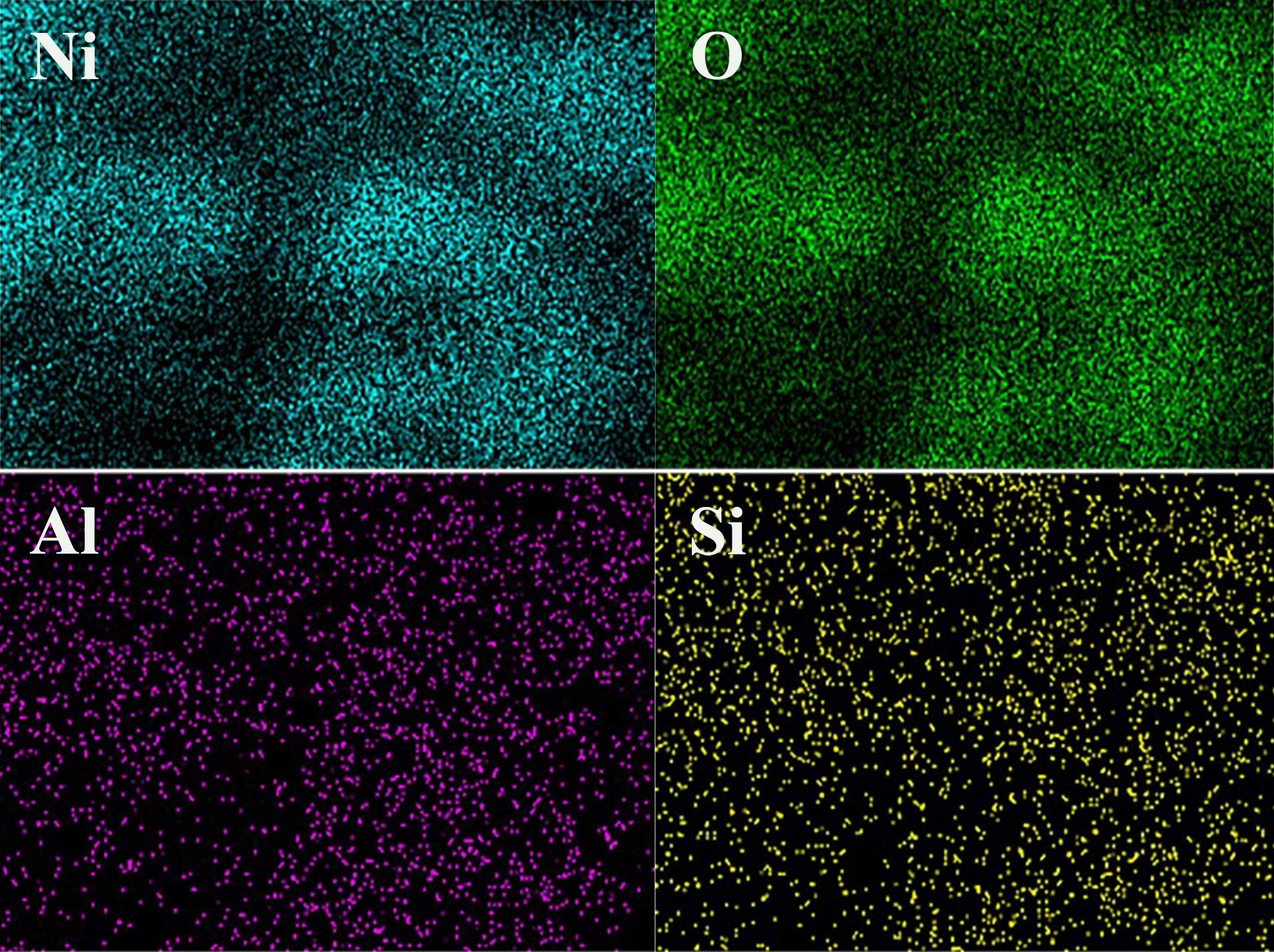

Supplement: Supplementary file 2 [file Image1.TIF]
